# A novel cartilage-targeting MOF-HMME-RGD sonosensitizer combined with sonodynamic therapy to enhance chondrogenesis and cartilage regeneration

**DOI:** 10.3389/fbioe.2024.1339530

**Published:** 2024-02-01

**Authors:** Shanchao Luo, Yifeng Shang, Zainen Qin, Bo Zhou, Chun Lu, Yangyang Qu, Jinmin Zhao, Ruiming Liang, Li Zheng, Shixing Luo

**Affiliations:** ^1^ Guangxi Engineering Center in Biomedical Materials for Tissue and Organ Regeneration, International Joint Laboratory on Regeneration of Bone and Soft Tissues, Guangxi Key Laboratory of Regenerative Medicine, Collaborative Innovation Center of Regenerative Medicine and Medical Bioresource Development and Application Co-constructed by the Province and Ministry, The First Affiliated Hospital of Guangxi Medical University, Nanning, Guangxi, China; ^2^ Department of Orthopaedics Trauma and Hand Surgery, The First Affiliated Hospital of Guangxi Medical University, Nanning, Guangxi, China; ^3^ Department of Orthopedics, The Ninth Affiliated Hospital of Guangxi Medical University, Guangxi Medical University, Nanning, Guangxi, China; ^4^ School of Materials and Environment, Guangxi Minzu University, Nanning, Guangxi, China

**Keywords:** articular cartilage regeneration, stem cell therapy, metal-organic framework, sonosensitizers, sonodynamic therapy

## Abstract

Articular cartilage regeneration is still a difficult task due to the cartilage’s weak capacity for self-healing and the effectiveness of the available therapies. The engineering of cartilage tissue has seen widespread use of stem cell-based therapies. However, efficient orientation of line-specific bone marrow mesenchymal stem cells (BMSCs) to chondrogenesis and maintenance of chondrogenic differentiation challenged stem cell-based therapy. Herein, we developed a Fe-based metal-organic framework (MOF) loaded with hematoporphyrin monomethyl ether (HMME) and cartilage-targeting arginine-aspartate-glycine (RGD) peptide to form MOF-HMME-RGD sonosensitizer to regulate BMSCs chondrogenic differentiation for cartilage regeneration via the modulation of reactive oxygen species (ROS). By using sonodynamic therapy (SDT), the MOF-HMME-RGD demonstrated favorable biocompatibility, could generate a modest amount of ROS, and enhanced BMSCs chondrogenic differentiation through increased accumulation of glycosaminoglycan, an ECM component specific to cartilage, and upregulated expression of key chondrogenic genes (*ACAN*, *SOX9*, and *Col2a1*). Further, transplanted BMSCs loading MOF-HMME-RGD combined with SDT enhanced cartilage regeneration for cartilage defect repair after 8 weeks into treatment. This synergistic strategy based on MOF nanoparticles provides an instructive approach to developing alternative sonosensitizers for cartilage regeneration combined with SDT.

## 1 Introduction

Articular cartilage, a thin tissue layer, effectively distributes loads by its unique extracellular matrix components, including collagen and chondroitin sulfate. This structural composition serves to safeguard the subchondral bone from excessive stress. (Gao et al., 2021). Due to its aneural, avascular, and alymphatic nature, severe cartilage damage or trauma is difficult to self-repair (Dai et al., 2015; Liu et al., 2021). The stem cell-based treatment using multipotent stem cells, which can be guided towards chondrogenic development, has found extensive use in the field of cartilage tissue engineering (Gugjoo et al., 2016; Lee and Wang, 2017). However, the efficient orientation of line-specific BMSCs to chondrogenesis and maintenance of chondrogenic differentiation challenged stem cell-based therapy (Shi et al., 2019).

It has been reported that moderate ROS is important for cell growth and differentiation. During the process of chondrogenesis, the involvement of ROS is of significant importance as they actively contribute to the stimulation of cell proliferation and the facilitation of differentiation in BMSCs while simultaneously preventing the onset of an inflammatory response (Kim et al., 2010; Lu et al., 2019; Wu et al., 2023a). Previous studies showed that the ROS level was specifically high in developing chondrocytes of the embryonic limb, and chondrocyte maturation was accompanied by a progressive decrease in catalase activity in growing cartilage (Salas-Vidal et al., 1998; Schnabel et al., 2006). Regarding *in vitro* chondrogenic differentiation, ROS generation was increased during induction (Jallali et al., 2007; Heywood and Lee, 2017). In addition, varieties of nanomaterials and hydrogels were capable of effectively promoting the chondrogenic differentiation of BMSCs due to the increasing generation of ROS (Lu et al., 2019; Cai et al., 2020; Wu et al., 2023b). Sonodynamic therapy (SDT) is a promising therapeutic modality that utilizes ultrasound (US) to induce sonoluminescence. This process activates sonosensitizers, leading to the generation of ROS, including hydroxyl radicals and singlet oxygen. SDT represents an ultrasound-based approach for the treatment of cartilage-related conditions, offering a new paradigm in the field of cartilage therapy (Uddin et al., 2016; Truong Hoang et al., 2022). The use of sonosensitizer-enabled SDT has been shown to effectively enhance the repair of articular cartilage lesions (Wu et al., 2023b). This approach has many advantages, including non-invasiveness, the ability to penetrate deep into the tissue, high therapeutic efficacy, and little occurrence of adverse effects (Vahedi et al., 2021). For SDT, the selection of appropriate sonosensitizers is critical.

Organic sonosensitizers like porphyrins, phthalocyanines, fluoroquinolone (FQ) antibiotics, phenothiazine compounds, *etc.*, with special groups producing sonosensitive activity by the US, have attracted much attention because of their easy monitoring, clear structure, stable chemical and physical properties, and evaluation of drug metabolism (Li et al., 2021). In particular, the porphyrins derivatives hematoporphyrin monomethyl ether (HMME) which is a second-generation hematoporphyrin (HP)-related sensitizer can produce a sensitive reaction to convert oxygen into ROS under US irradiation and has been continuously used as US-responsive sonosensitizer in SDT with the advantage of high quantum yield in the ROS generation, good selective retention and long-term retention in tissue (Huang et al., 2018; Li et al., 2021; Song et al., 2023). Furthermore, HMME was a hydrolysis product of endogenous hemoglobin with good biocompatibility, sonosensitizing ability, and emissive properties. Previous research has confirmed that HMME has been widely used in SDT by US irradiation for medication of ROS-related diseases, including antitumor treatment, sonodynamic antimicrobial chemotherapy, and neovascularization theranostics. It can easily control the amount of ROS production by regulating the concentration of HMME and several the sonodynamic parameters including ultrasonic intensity, irradiation duration and frequency. Wen et al. successfully synthesized polymeric nanoparticles, namely, CAT@HA-HMME NPs, whereby HMME was used as a representative organic sonosensitize (Wen et al., 2022). These nanoparticles were designed to exhibit prolonged blood circulation and disintegration inside tumor sites, hence enhancing the efficacy of sonodynamic treatment. Yao et al. constructed ferrite-encapsulated nanoparticles that are sensitive to low-intensity focused ultrasoun (Yao et al., 2021). These nanoparticles were created via HMME-mediated SDT and were designed for theranostics targeting neovascularization in atherosclerotic plaques. Xu reported the synthesis of Fe-HMME coordination particles (FeCPs) by the assembly of HMME with Fe (III) ions (Xu et al., 2020). These particles were then loaded with the anticancer medication doxorubicin to enable a combination therapy approach using SDT and chemotherapy for the treatment of deep-seated tumors. Nonetheless, HMME-based nanoparticles remain unsatisfactory because of poor water solubility, poor light stability, low efficient delivery, rapid metabolism, fast clearance, *etc.*, resulting in reduced bioavailability and restricted absorption into the body (Huang et al., 2018; Wen et al., 2022). These aforementioned limitations give rise to suboptimal pharmacokinetics and a lack of therapeutic efficacy in the context of SDT. Hence, the development of novel techniques is important to get optimal SDT using organic sonosensitizers.

Metal-organic frameworks (MOFs) are a category of porous coordination polymers that have garnered significant attention due to their growing and practical applications. The formation of coordination bonds is facilitated by the coordination of metal ions or clusters with functional organic ligands, resulting in the creation of these frameworks (Wen et al., 2023). MOFs have attracted extensive research interest and have been widely used in drug delivery due to the unique features of facile synthesis, diverse compositions, adjustable porosity, high surface areas, easy surface functionalization, good dispersibility, high loading capacities, and tunable biocompatibility (Jiang et al., 2023). In our previous studies, we developed pH-responsive MOFs modified by hyaluronic acid (HA) loaded with anti-inflammatory protocatechuic acid (PCA) for osteoarthritis therapy (Xiong et al., 2020) and zinc-based MOFs with antibacterial and anti-inflammatory properties for promoting wound healing (Chen et al., 2022). It was found that the porous structure of MOFs can prevent the self-quenching of organic acoustic molecules and promote the rapid diffusion of ROS, thus increasing the quantum yield of ROS by leveraging the benefits offered by the linker-to-metal charge transfer mechanism (Xie et al., 2022). Thus, MOFs may be promising carriers for HMME in SDT applications.

**SCHEME 1:**
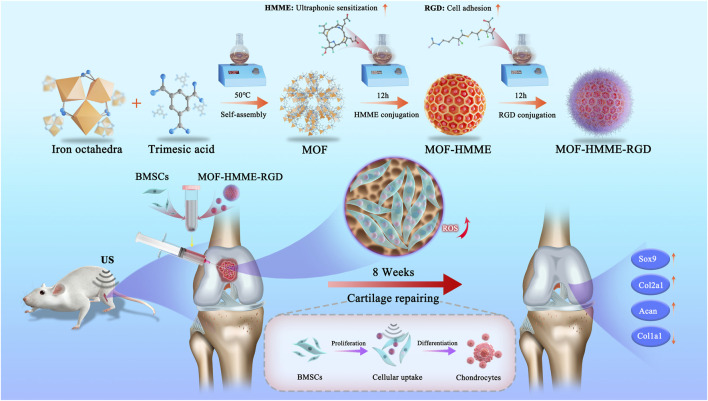
Schematic illustrations of MOF-HMME-RGD synthesis and the potential therapeutic mechanisms of the multifunctional combined strategy.

Herein, we developed MOF-based multifunctional therapeutic nanoparticles loading with HMME and arginine-aspartate-glycine (RGD) (MOF-HMME-RGD) with a combination of SDT for cartilage regeneration in an attempt to regulate chondrogenic differentiation of BMSCs by producing moderate ROS (Scheme 1). HMME can serve as sonosensitizers to produce ROS (e.g., singlet oxygen) via US stimulation. The RGD peptide is well recognized as a particular binding site for integrin receptors, which are key regulators of cell-extracellular and cell-cell microenvironment communication (Koh et al., 2022). Therefore, it is anticipated that MOF-HMME-RGD would enhance cell viability, stimulate the production of proteoglycans, and facilitate the restoration of cartilage by expediting the lineage-specific differentiation of BMSCs towards chondrogenic lineage and cartilage regeneration. This strategy based on MOF nanoparticles provides an instructive approach to developing alternative sonosensitizers for cartilage regeneration combined with SDT.

## 2 Materials and methods

### 2.1 Synthesis of MOF-HMME-RGD nanoparticles (NPs)

#### 2.1.1 Preparation of metal-organic frameworks (MOFs) NPs

Metal-organic frameworks nanoparticles were synthesized utilizing a technique that was derived from prior research, with some adjustments made to the procedure (Cai et al., 2017). A mixture was prepared by combining 40 mL of a 2 mM FeCl_3_ solution (dissolved in methanol) with 40 mL of 1.2 mM 1, 3, 5-benzene tricarboxylic acid (trimesic acid, BTC) solution (also dissolved in methanol). The mixture was subjected to vigorous stirring for 10 min. The solution was put into a heated oil bath maintained at a temperature of 50°C and allowed to undergo a reaction for 90 min. Subsequently, the solution underwent centrifugation for 12 min at a rotational speed of 12,000 revolutions per minute (rpm) using a centrifuge (Eppendorf 5810R, Germany). Following centrifugation, the resulting pellet was subjected to three consecutive washes with deionized water. The MOFs NPs were ultimately acquired by the process of freeze-drying in a freeze dryer and kept at a temperature of 4°C.

#### 2.1.2 Preparation of modified MOF-HMME NPs

5 mL MOF methanol solution (1.0 mg/mL) and 5 mL HMME methanol solution (1.0 mg/mL) were mixed under ultrasonic conditions. Then, the aforementioned solution was subjected to agitation at ambient temperature for 12 h. Subsequently, the obtained mixtures were subjected to centrifugation at a rotational speed of 10,000 rpm for 5 min, and followed by a triple rinsing procedure using ethanol and deionized water. The resultant product was obtained via the process of centrifugation and then designated as MOF-HMME. Thereafter, the resulting product was reconstituted by adding 5 mL ultrapure water and stored at 4°C.

### 2.2 Preparation of modified MOF-HMME-RGD NPs

The methanol solution containing 5 mg MOF-HMME NPs and the solution containing 0.5 mg RGD (5 mg/mL) were mixed under sonication conditions and placed at a constant temperature of 37°C for 12 h in a shaker. The MOF-HMME-RGD was obtained by centrifugation to remove the unloaded RGD. Finally, MOF-HMME-RGD NPs were resuspended with 5 mL ultrapure water and stored at 4°C.

### 2.3 Characterization of MOF-HMME-RGD

The morphology and structure of MOF-HMME-RGD nanoparticles were examined using transmission electron microscopy (TEM) and X-ray diffraction (XRD) techniques, respectively. The TEM analysis was conducted using a Hitachi instrument from Japan, while the XRD analysis was performed using a MiniFlex 600 instrument, also from Japan. The absorption spectra of nanoparticles (NPs) in phosphate-buffered saline (PBS) were measured using a UV-visible spectrophotometer (Shimadzu, UV-2700, Japan). The average particle size and zeta potential of nanoparticles in a PBS solution were measured using Nano-ZS equipment manufactured by Malvern Panalytical in China. The HMME releasing behaviors from MOF-HMME-RGD were investigated. 200 μg MOF-HMME-RGD was dissolved in 1 mL of PBS buffer and then placed at 37°C with or without ultrasound stimulation under thermostatic shaking. The HMME concentration in the buffer was measured uniformly using a UV spectrophotometer at 0, 10, 30, 60, 120, 180 min, and the drug release rate (Q) was further calculated.

$$Q\left(\%\right)=\left(C_{t}V_{0}+\sum_{i=1}^{t-1}C_{i}V_{i}\right)/W\times 100$$

C_t_ and C_i_ in equation represent the concentration of HMME in the *t*th and *i* ^th^ collected samples, respectively. V_0_ and V_i_ represent the original volume in the simulated system and the volume of the *i* ^th^ sampling, respectively (V_0_ = 1 mL; V_i_ = 1 mL), and W is the total amount of drug HMME in the simulated system.

### 2.4 Detection of singlet oxygen

The single oxygen generated by MOFs, HMME, MOF-HMME, MOF-HMME-RGD was measured by a chemical probe 1, 3-diphenylisobenzofuran (DPBF). Typically, 10 μL 10 mM DPBF ethanol solution was added to 1.0 mL of PBS, and MOFs, HMME, MOF-HMME, and MOF-HMME-RGD were immersed into the above solution under ultrasound stimulation with a frequency of 1.0 MHz, a sound intensity of 0.35 W/cm^2^, a duty cycle of 60%. Finally, the absorbance of DPBF at a wavelength of 410 nm was assessed using a microplate reader.

### 2.5 Cell isolation and culture

The animal experiments conducted in this study were approved by the Animal Ethics Committee standards of Guangxi Medical University, by Protocol Number 201903034. The animals were euthanized with the administration of sodium pentobarbital. BMSCs were obtained from the femoral marrow of neonatal Sprague-Dawley (SD) rats, as described in previous work. (Gao et al., 2023). BMSCs were cultivated in α-minimal essential medium (α-MEM, Biosharp, China) with 1% penicillin/streptomycin antibiotics (Biosharp, China) and 10% fetal bovine serum (Tianhang, China). The cells were subjected to incubation at a temperature of 37°C under an atmosphere containing 5% carbon dioxide. The medium was replenished at regular intervals of every 3 days.

### 2.6 Intracellular ROS detection

The measurement of intracellular ROS generation was conducted using a probe kit containing 2′,7′-dichlorofluorescein diacetate (DCFH-DA) obtained from Thermo Fisher Scientific, located in the United States. The BMSCs were cultured in 6-well plates with a density of 2 × 10^5^ cells per well. The fluorescent probe DCFH-DA can enter cells and undergo cleavage upon oxidation by ROS. Subsequently, it is de-esterified by endogenous esterases and transformed into the fluorescent molecule 2′,7′-dichlorofluorescein (DCF). In summary, the cells that had undergone treatment were gathered and reconstituted with a diluted solution of DCFH-DA (10 μM). Subsequently, the cells had an incubation phase of 20 min at a temperature of 37°C inside a regulated cell incubator. The cells underwent centrifugation, followed by three rounds of washing, and were then resuspended using a serum-free cell culture medium. The quantification of ROS inside the intracellular environment was conducted utilizing a fluorescence spectrophotometer. The excitation wavelength used was 488 nm, while the emission wavelength utilized was 525 nm.

### 2.7 Cell viability and cell apoptosis assay

The optimal concentration of HMME incorporated in MOF-HMME-RGD, along with the ultrasonic intensity, irradiation duration, and frequency, were determined based on the results obtained from the cell viability analysis performed using the 3-(4,5-dimethylthiazol-2-yl)-2,5-diphenyltetrazolium bromide (MTT) assay provided by Gibco, United States. The BMSCs were cultured in 6-well plates with a density of 2×10^5^ cells per well. The absorbance value at 490 nm was measured using the microplate reader from Thermo Fisher Scientific, United States. Subsequently, the computation of cell viability was conducted. The treatment parameters which the most substantial support for cells proliferation were chosen for the subsequent experiment. The experiment was divided into five groups: (1) Control group; (2) T group: BMSCs were cultured in the chondrogenic medium; (3)TU group: BMSCs were cultured in chondrogenic medium with ultrasound stimulation; (4)TMU group: BMSCs were cultured in chondrogenic medium containing MOF-HMME-RGD (C_HMME_ = 1 μg/mL) with ultrasound stimulation; (5) Nac-TMU group: BMSCs were cultured in chondrogenic medium containing MOF-HMME-RGD (C_HMME_ = 1 μg/mL) and NAC (a ROS scavenger) with ultrasound stimulation. The cultured BMSCs were carried out with ultrasound stimulation by a medical ultrasound physiotherapy instrument (WELLD, China). The treatment parameters of LIPUS were: free mode, switching ratio of 60%, frequency of 1 MHz, ultrasound intensity of 0.35 W/cm^2^, irradiation time of 60 s, and irradiation frequency of once a day. The apoptosis of the BMSCs with ultrasound stimulation was determined with the Annexin V-EGFP/PI apoptosis kit (KeyGEN BioTECH, Nanjing, China) by a flow cytometer (BD FACSCalibur, USA).

### 2.8 Intracellular uptake of MOF-HMME-RGD

The BMSCs were cultured in 6-well plates with a density of 3×10^4^ cells per well. After 12 h of incubation, the cells were cultured with 3 mL medium containing FITC-labeled MOF-HMME-RGD (C_HMME_ = 1 μg/mL). Samples were collected and observed using fluorescence microscopy at 4h, 1d, and 3d.

### 2.9 Biochemical assays

To further explore the synergistic effect of MOF-HMME-RGD on BMSCs chondrogenesis with SDT, the cells were cultured in chondrogenic medium containing 10 ng mL^−1^ transforming growth factor-*β*1 (TGF-*β*1, PeproTech, USA), 50 μg mL^-1^ ascorbic acid (Solarbio, China), 50 mg mL^-1^ insulin-transferrin-selenium (Gibco, USA), 100 nmol mL^−1^ dexamethasone (Solarbio, China) at 5% CO_2_, 37°C for 14 days. The BMSCs were cultured in 6-well plates with a density of 2×10^5^ cells per well. The experiment was divided into five groups: (1) Control group; (2) T group: BMSCs were cultured in the chondrogenic medium; (3)TU group: BMSCs were cultured in chondrogenic medium with ultrasound stimulation; (4)TMU group: BMSCs were cultured in chondrogenic medium containing MOF-HMME-RGD (C_HMME_ = 1 μg/mL) with ultrasound stimulation; (5) Nac-TMU group: BMSCs were cultured in chondrogenic medium containing MOF-HMME-RGD (C_HMME_ = 1 μg/mL) and NAC (a ROS scavenger) with ultrasound stimulation. The treatment parameters of LIPUS were: free mode, switching ratio of 60%, frequency of 1 MHz, ultrasound intensity of 0.35 W/cm^2^, irradiation time of 60 s, and irradiation frequency of once a day.

The cells were subjected to a series of procedures at each time point. Firstly, they were washed three times with a PBS solution. Subsequently, the cells were digested with a 1.0 mL solution of proteinase K, which had a concentration of 20 mg/mL. This digestion process took place at a temperature of 56°C for 10 h. The purpose of these procedures was to quantify the levels of DNA and glycosaminoglycan (GAG) present in the cells. DNA contents were measured using the Hoechst 33,258 dye with DNA from the calf thymus as a standard. The GAG contents were quantified using dimethyl methylene blue (DMMB) chloride methods, and optical density was tested at 525 nm. The quantification of GAG secretion was performed using a calibration curve based on the absorbance of chondroitin sulfate, and the results were normalized to account for the total DNA content.

### 2.10 Quantitative real-time polymerase chain reaction (qRT-PCR) analysis

Quantitative real-time polymerase chain reaction (qRT-PCR) was used to evaluate the expression levels of cartilage-specific genes (*Acan*, *Sox9*, *Col2a1*, *Col1a1*) in BMSCs. The BMSCs were cultured in 6-well plates with a density of 2 × 10^5^ cells per well in chondrogenic medium for 14 days. The treatment parameters of LIPUS were: free mode, switching ratio of 60%, frequency of 1 MHz, ultrasound intensity of 0.35 W/cm^2^, irradiation time of 60 s, and irradiation frequency of once a day. The process of reverse transcription (RT) was conducted to convert total RNA into complementary DNA (cDNA) using a Prime Script RT kit manufactured by Takara in Japan. The qRT-PCR experiment was performed using the Fast Start universal SYBR Green Master Mix (Roche, Germany) on a detection system. The PCR cycling protocol included an initial denaturation step at a temperature of 95°C for 180 s, followed by a series of 40 cycles, including denaturation at 95°C for 3 s and annealing at 60°C for 30 s. The primer sequences for both the forward and reverse directions were documented in Table 1. The quantification of mRNA expression levels was conducted by using GAPDH as an internal control and using the threshold cycle (Ct) technique to determine the values represented as R = 2^−ΔΔCT^.

**TABLE 1:** Primers in qRT-PCR analysis.

Genes	Primers	Sequences
*GAPDH*	Forward	AGG​CCG​GTG​CTG​AGT​ATG​TC
	Reverse	TGC​CTG​CTT​CAC​CAC​CTT​CT
*Acan*	Forward	CCG​CTG​GTC​TGA​TGG​ACA​CT
	Reverse	AGG​TGT​TGG​GGT​CTG​TGC​AA
*Col2α1*	Reverse	TGC​CTG​CTT​CAC​CAC​CTT​CT
	Reverse	ACC​CCT​CTC​TCC​CTT​GTC​AC
*Col1α1*	Forward	GAG​AGG​TGA​ACA​AGG​TCC​CG
	Reverse	AAA​CCT​CTC​TCG​CCT​CTT​GC
*SOX9*	Forward	TCG​GTG​AAG​AAT​GGG​CAA​GC
	Reverse	CTG​AGA​TTG​CCC​GGA​GTG​CT

### 2.11 Animal procedure

The animal experiments conducted in our research were conducted according to the ethical guidelines established by the Animal Ethics Committee of Guangxi Medical University (Protocol Number: 201,903,034). In this investigation, a cohort of 24 Sprague-Dawley rats, aged between 8 and 10 weeks and weighing around 200 g, was used. Following the administration of general anesthesia using a 2% solution of sodium pentobarbital at a dosage of 30 mg/kg, a controlled experiment was conducted to create a defect only inside the cartilage tissue. The observed defect had a diameter of 3 mm and a depth of 2 mm, and it was located inside the articular cartilage area of the distal femoral condyle in the knee joint. The Sprague-Dawley rats were allocated into four groups using a random assignment method. The study consisted of four experimental groups: (1) Control group: untreated cartilage defect model, (2) BMSCs group: treated with implantation of 10 × 10 ^6^ BMSCs, (3) BMSCs + US group: treated with implantation of 10 × 10 ^6^ BMSCs and ultrasound stimulation, (4) SDT group: treated with implantation of 10 × 10 ^6^ BMSCs uptaking MOF-HMME-RGD NPs and ultrasound stimulation. The treatment parameters of LIPUS were: free mode, switching ratio of 60%, frequency of 1 MHz, intensity of 0.35 W/cm^2^, 1 min per exposure, and one exposure every other day.

### 2.12 Gross observation, histological staining, and grading

The femoral condyle’s articular cartilage, which had undergone repair, was collected for macroscopic examination and grading at two specific time intervals: 4 weeks and 8 weeks after the surgical intervention. A morphological evaluation of cartilage regeneration was performed by three impartial assessors who were blinded to the experimental groups. The evaluation was completed in accordance with the grading method established by the International Cartilage Repair Society (ICRS).

After doing a first visual inspection, the femoral condyles were fixed by immersing them in a 4% paraformaldehyde solution for 48 h. Subsequently, decalcification was carried out using an aqueous solution with a pH of 7.2, consisting of 10% (v/v) ammonium hydroxide and 14% (w/v) EDTA. This decalcification process was facilitated by an ultrasonic machine manufactured by Medite in Germany and lasted for 2 weeks. Following the process of dehydration using graded ethanol, the samples underwent paraffin embedding and were then sectioned into slices measuring 3 mm in thickness. The samples underwent staining using safranin O/fast green and the hematoxylin-eosin (HE) kit (Solarbio, Peking, China). The immunohistochemical staining technique was used to analyze the expression of *Col2a1* using a specific *Col2a1* antibody (Bioss, Peking, China) and the Biotin-Streptavidin HRP Detection System. The specimens were visually examined and collected using an optical microscope (BX 63, Olympus, Japan). The articular cartilage that underwent repair in each experimental group was evaluated and rated using the Modified O'Driscoll Score (MODS). Three independent observers who were unaware of the research settings, assessed the histological evaluation scale. (Betz et al., 2017).

### 2.13 Statistical analysis

The data underwent analysis using SPSS 22.0 (SPSS Inc., Chicago, Illinois, USA) and were afterward published as mean ± standard deviation. The statistical significance between groups was assessed using a significance level of *p* < 0.05. The study used a one-way analysis of variance (ANOVA) to investigate the differences between two cohorts, followed by a *post hoc* analysis using the Least Significant Difference (LSD) approach.

## 3 Results

### 3.1 Preparation and characterization of MOF-HMME-RGD

**FIGURE 1:**
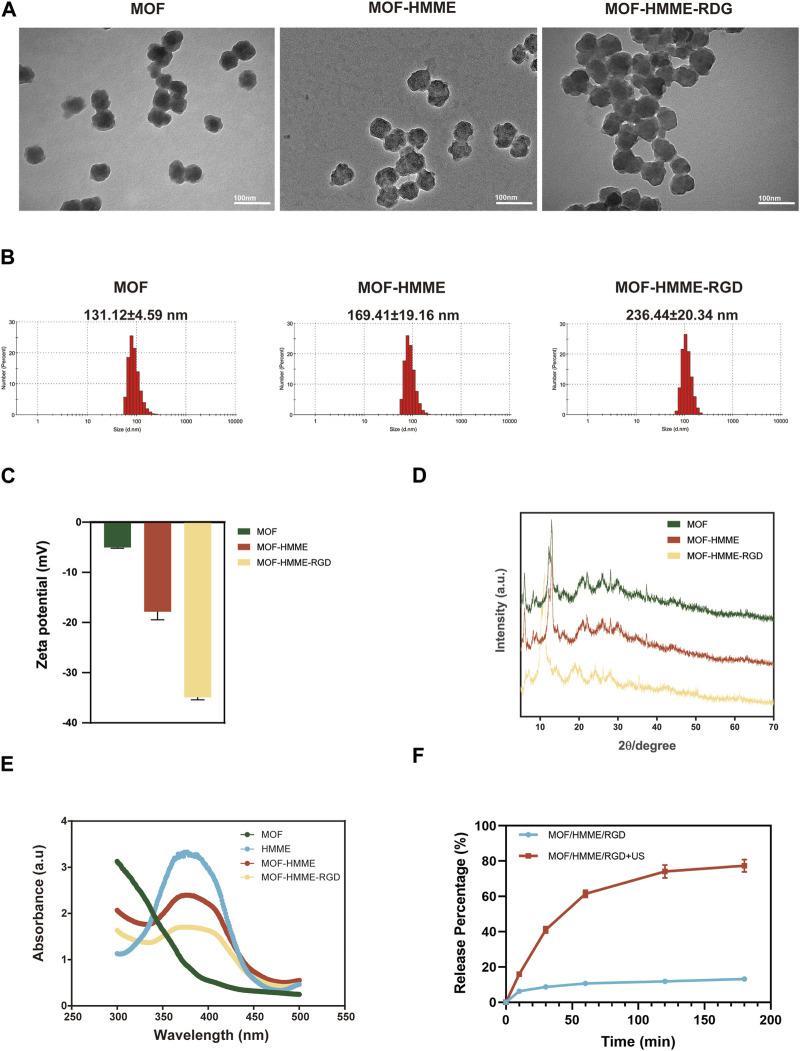
The characterizations of MOF-HMME-RGD. **(A)** TEM images, scale bar = 100 nm. **(B)** Particle size and **(C)** zeta potentials were tested by a Malvern particle size analyzer. **(D)** X-ray diffraction (XRD) analysis. **(E)** UV spectra of MOF-HMME-RGD. **(F)** The HMME releasing behaviors from MOF-HMME-RGD.

The synthesis of the multifunctional MOF-HMME-RGD was carried out according to the methodology depicted in Scheme 1. We obtained MOF-HMME-RGD by stepwise stirring reaction under mild conditions. The compound [Fe (BTC)] (BTC: 1,3,5-benzene dicarboxylate) consists of clusters of iron octahedra that are connected by a shared vertex μ3-O, with the connection facilitated by the BTC moieties. The TEM images (Figure 1A) revealed that the MOF, MOF-HMME, and MOF-HMME-RGD samples exhibited a distinct spherical morphology with uniform size distribution. The mean diameters of the MOF, MOF-HMME, and MOF-HMME-RGD were 131.12 ± 4.59 nm,169.41 ± 19.16 nm, and 236.44 ± 20.34 nm (Figure 1B), respectively, which indicates that all three samples exhibited a reasonably narrow size distribution, with no statistically significant differences observed between them. In addition, the average zeta potentials of MOF, MOF-HMME, and MOF-HMME-RGD were −5.03 ± 0.23 mV, −17.87 ± 2.22 mV and −34.93 ± 0.67 mV (Figure 1C), respectively. Figure 1D presented the XRD pattern of the MOF, MOF-HMME, and MOF-HMME-RGD. The X-ray diffraction patterns exhibited distinct diffraction peaks, all of which were successfully attributed to a cubic crystal structure. This observation aligns with the findings documented in previous studies. The introduction of HMME and RGD did not induce any alterations in the crystalline structure of Fe-BTC nanoparticles (metal-organic framework). In order to conduct a more comprehensive examination of the optical characteristics, UV-visible spectroscopy was employed to ascertain the loading quantities of HMME within the MOF NPs. The absorption spectra of MOF, MOF-HMME, and MOF-HMME-RGD were shown in Figure 1E. The MOFs had no obvious photoabsorption in a wide range (300–500 nm). The MOF-HMME and MOF-HMME-RGD NPs had maximum absorption peaks at 390 nm after the incorporation of HMME due to π-π stacking, and loading RGD did not affect the absorption peak of MOF-HMME-RGD. The HMME releasing behaviors from MOF-HMME-RGD were investigated in Figure 1F. The HMME releasing from MOF-HMME-RGD under ultrasonic stimulation reached the maximum in 180 min and the cumulative release was 77.41%, while that in the MOF-HMME-RGD without ultrasonic stimulation was 13.18%.

### 3.2 Cell viability assessment of MOF-HMME-RGD

**FIGURE 2:**
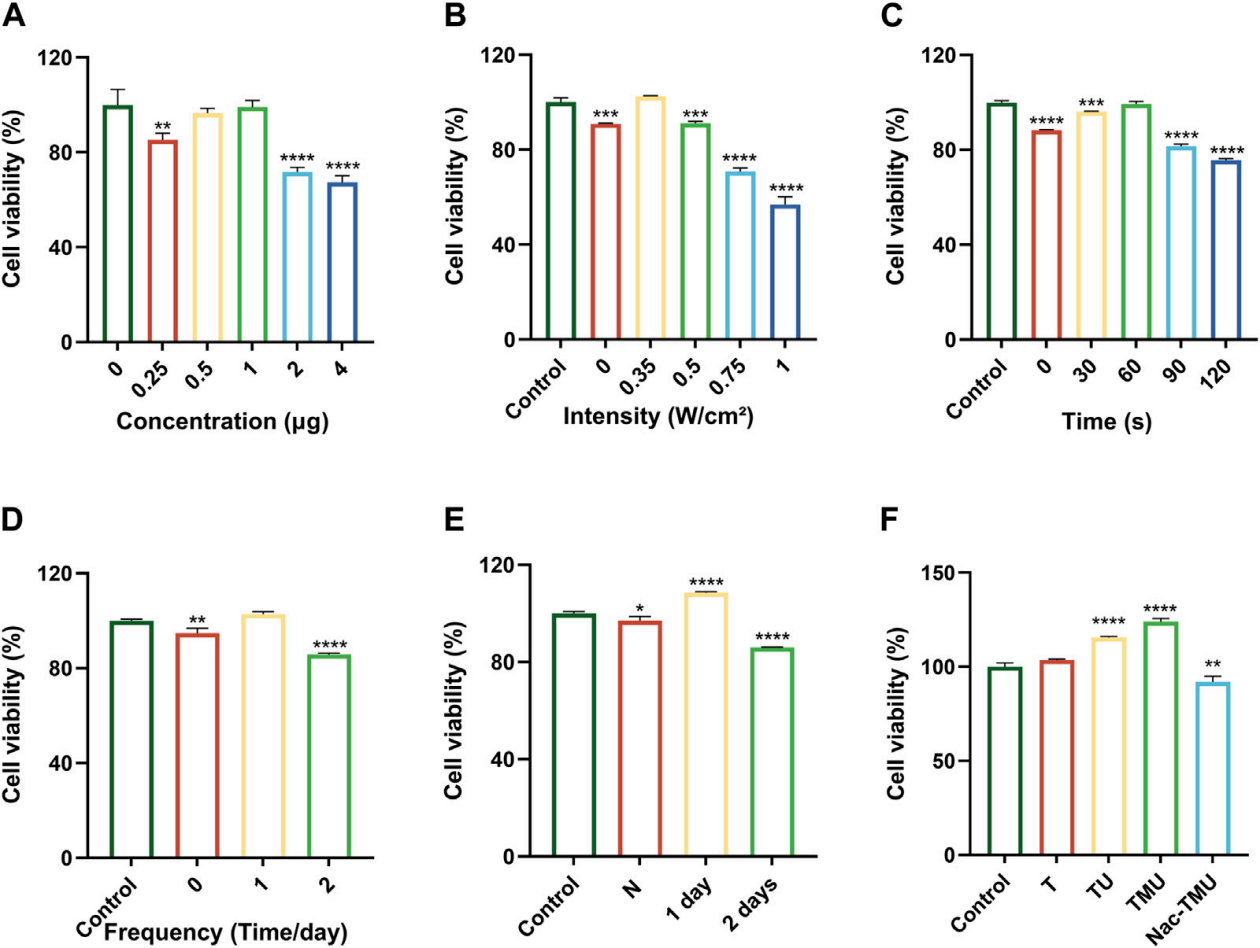
Cell viability assay **(A)** Viability of BMSCs cultured in different concentrations of HMME loaded in MOF-HMME-RGD for 24 h. **(B)** Viability of BMSCs with different ultrasound intensities. **(C)** Viability of BMSCs with different ultrasound times (0.35 W/cm^2^ ultrasound intensity). **(D)** Viability of BMSCs with different irradiation frequencies once a day. **(E)** Viability of BMSCs at different ultrasound intervals. **(F)** Viability of BMSCs cultured in optimal conditions. Mean ± SD, n = 3. * symbol compared with control group, ^**^ *p* < 0.01, ^***^ *p* < 0.001, and ^****^ *p* < 0.0001.

The determination of the ideal concentration of HMME loaded in MOF-HMME-RGD, as well as the ultrasonic intensity, irradiation time, and frequency, was based on the results of cell viability assessed via the MTT assay. The cellular viability exhibited an upward trend as the concentration of HMME encapsulated within MOF-HMME-RGD remained below 1.0 μg, after which they decreased significantly (Figure 2A). For the optimal treatment time, the cellular viability was increased when the ultrasound intensity was maintained at 0.35 W/cm^2^ (Figure 2B), and the irradiation time was less than 60 s (Figure 2C), which dropped considerably afterward. When different ultrasound frequencies were performed each day, there was the highest cell viability when cells were treated with ultrasound once a day (Figure 2D), and cell viability was greatly reduced in 2 days of irradiation compared to only 1 day of irradiation (Figure 2E). Thus, ultrasound intensity of 0.35 W/cm^2^, irradiation time of 60 s, and irradiation frequency of once a day, which provided the most substantial support for the proliferation of cells, were chosen for the subsequent experiment.

As shown in Figure 2F, the TMU group had the highest cell viability under ultrasound stimulation, and cell viability was significantly reduced after the addition of ROS scavenger NAC in the Nac-TMU group. This result indicated that the MOF-HMME-RGD with ultrasound stimulation significantly increased the cell viability.

### 3.3 Intracellular ROS generation of MOF-HMME-RGD

**FIGURE 3:**
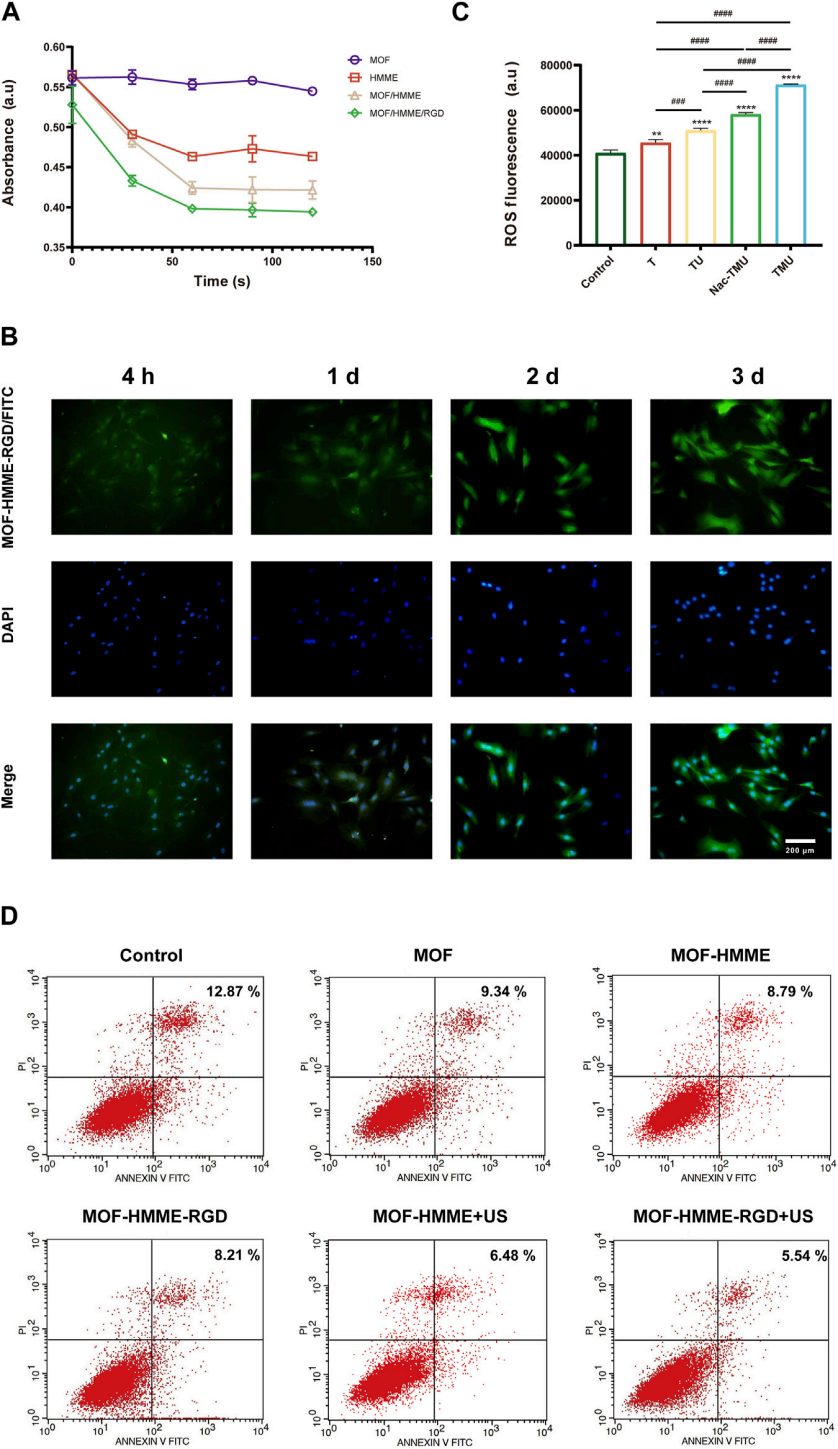
ROS generation of MOF-HMME-RGD. **(A)** The capability of singlet oxygen generation was tested by a chemical probe DPBF under different ultrasound times. **(B)** Cellular uptake of MOF-HMME-RGD (MOF-HMME-RGD/FITC: green, DAPI: blue). (Scale bar = 200 μm). **(C)** Intracellular ROS production was assessed with a DCFH-DA probe. **(D)** The apoptosis of the BMSCs with ultrasound stimulation was determined by flow cytometry. Mean ± SD, n = 3. * symbol compared with control group, ^**^ *p* < 0.01, ^***^ *p* < 0.001, and ^****^ *p* < 0.0001, and # symbol compared between groups, ^#^ *p* < 0.05, ^##^ *p* < 0.01, ^###^ *p* < 0.001, and ^####^ *p* < 0.0001.

To explore the source of ROS, the capability of singlet oxygen generation by MOF-HMME-RGD was detected by a chemical probe 1, 3-diphenylisobenzofuran (DPBF) at 410 nm. As illustrated in Figure 3A, the production level of singlet oxygen in MOF-HMME-RGD and MOF-HMME is much higher than that in MOF when irradiated by ultrasound, as demonstrated by the characteristic absorption peak of DPBF decreased with the increase of irradiation time. Besides, the decrease in absorbance of the MOF-HMME-RGD group was quicker than that of the MOF-HMME group, which may be due to the synergistic effect of HMME and RGD to produce more singlet oxygen. In contrast, MOF cannot decline the absorption of DPBF, indicating that MOFs are not able to generate singlet oxygen.

The cellular uptake behavior of MOF-HMME-RGD was investigated in BMSCs for 3 days. The nucleus was subjected to blue fluorescence staining using 4′,6-diamidino-2-phenylindole (DAPI), whereas the MOF-HMME-RGD was labeled with green fluorescence using FITC. As shown in Figure 3B, the cytoplasm exhibited a distribution of MOF-HMME-RGD at the 4-h time point, indicating the effective endocytosis of MOF-HMME-RGD by BMSCs.

During chondrogenesis, appropriate ROS are required for cell growth and differentiation of BMSCs. In order to verify the ROS generation capacity of MOF-HMME-RGD mediated by ultrasound, intracellular ROS production in BMSCs was measured using a chemical probe DCFH-DA. The compound DCFH-DA has the ability to undergo a reaction with ROS, resulting in the formation of 2′,7′-dichlorofluorescein (DCF). This reaction leads to the emission of green fluorescence. As shown in Figure 3C, there was lower fluorescence in the control group and the T group. However, the highest green fluorescence was found in the TMU group and decreased in the Nac-TMU group, which was treated with a ROS scavenger, the N-acetyl cysteine (NAC), indicating that the intracellular ROS can be produced by MOF-HMME-RGD nanoparticles and ROS generation capacity of MOF-HMME-RGD can be enhanced with ultrasound irradiation.

The apoptosis of the BMSCs with ultrasound stimulation was determined to investigate the effect of ROS generation by MOF-HMME-RGD nanoparticles on cell apoptosis. As shown in Figure 3D, the percentage of cell apoptosis in the MOF-HMME + US irradiation (6.48%) and MOF-HMME-RGD + US irradiation (5.54%) was lower than that in the MOF-HMME (8.79%) and MOF-HMME-RGD (8.21%), indicating ROS generation by MOF-HMME-RGD nanoparticles was not cause more apoptosis.

### 3.4 MOF-HMME-RGD combined with SDT promoted chondrogenic differentiation of BMSCs

**FIGURE 4:**
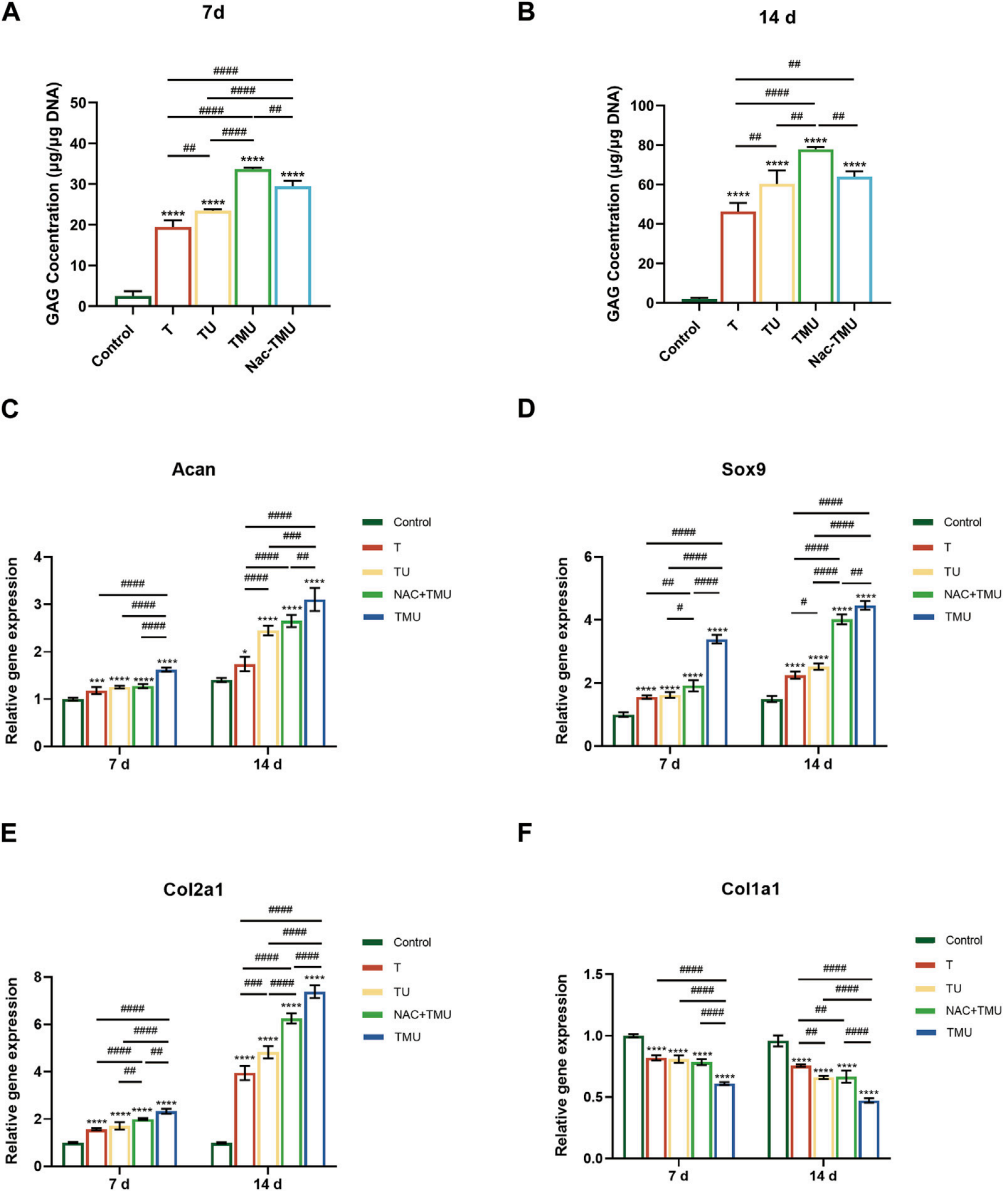
MOF-HMME-RGD combined with SDT promoted chondrogenic differentiation of BMSCs. **(A,B)** Secretion of GAG was evaluated by using DMMB assay for 7 days **(B)** and 14 days **(C)**. **(C–F)** The expression levels of *ACAN*, *SOX9*, *Col2a1*, and *Col1a1* in BMSCs were examined by qRT-PCR for 7 and 14 days. Mean ± SD, n = 3, * symbol compared with control group, ^**^ *p* < 0.01, ^***^ *p* < 0.001, and ^****^ *p* < 0.0001, and # symbol compared between groups, ^#^ *p* < 0.05, ^##^ *p* < 0.01, ^###^ *p* < 0.001, and ^####^ *p* < 0.0001.

The evaluation of GAG, a key component of the ECM present in cartilage, was conducted through the utilization of the DMMB test. As shown in Figure 4A and Figure 4B, MOF-HMME-RGD increased the GAG content via ultrasound by 73.1% and 68.4% on days 7 and 14 compared to the T group, respectively. However, GAG secretion was significantly decreased with the amount by 12.5% and 17.8% on days 7 and 14, respectively, in the Nac-TMU group treated with NAC. Thus, MOF-HMME-RGD could promote the secretion of GAG in BMSCs under ultrasound treatment.

To further explore the synergistic effect of MOF-HMME-RGD on BMSCs chondrogenesis with SDT, the cartilage-specific genes of BMSCs were examined by qRT-PCR (Figures 4C–F). The levels of chondrogenic markers *ACAN*, *SOX9*, and *Col2a1* exhibited a notable increase in the T, TU, Nac-TMU, and TMU groups as compared to the control group. This increase was more pronounced in the TMU group. However, the gene expression of fibrocartilage marker *Col1a1* was significantly downregulated in the TMU group.

### 3.5 MOF-HMME-RGD and BMSCs combined with SDT promoted cartilage defect repair *in vivo*

**FIGURE 5:**
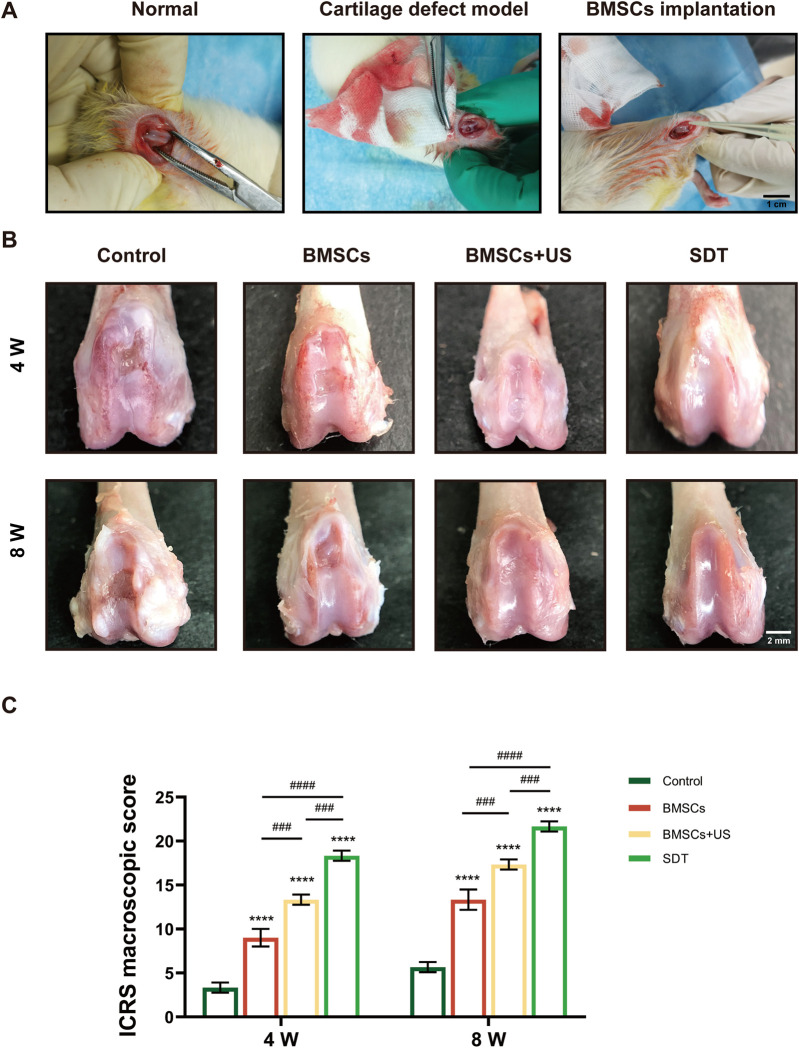
Macroscopic observation and scores of therapeutic effects of cartilage defect *in vivo*. **(A)** Animal operation at the articular cartilage site of the distal femoral condyle of the knee in adult SD rats. **(B)** Macroscopic observation of repaired cartilage after 4 weeks and 8 weeks of treatment. **(C)** The corresponding International Cartilage Repair Society (ICRS) macroscopic score. Mean ± SD, n = 3, * symbol compared with control group, ^**^ *p* < 0.01, ^***^ *p* < 0.001, and ^****^ *p* < 0.0001, and # symbol compared between groups, ^#^ *p* < 0.05, ^##^ *p* < 0.01, ^###^ *p* < 0.001, and ^####^ *p* < 0.0001.

In order to assess the potential for cartilage regeneration, a study was conducted using MOF-HMME-RGD and BMSCs as therapeutic agents to repair cartilage lesions of 3 mm × 3 mm×2 mm on the distal femoral condyle surface of Sprague-Dawley rats (Figure 5A). The cartilage regeneration was evaluated comprehensively after 4 weeks or 8 weeks of treatment, respectively. As shown in Figure 5B, there was nearly no inflammation, and synovial hyperplasia was observed for all implanted BMSCs compared to the control group, only with neo-cartilage tissue regenerated. Thin fibrous tissue with distinct borders was observed in the control group, indicating poor cartilage self-healing ability. Gross examination of knee joints (Figure 5C) showed that fibrous cartilage tissue formed within the defective area both in the BMSCs and BMSCs + US groups after 4 weeks and 8 weeks of repair. In contrast, smooth white repair tissue with indistinguishable borders could be observed in the SDT group, indicating a superior therapeutic effect. The ICRS macroscopic scores at 4 weeks and 8 weeks post-repair indicated that the SDT group had scores of 18.3 and 21.7, respectively. These values were found to be greater than those observed in the other groups. (Figure 5C).

**FIGURE 6:**
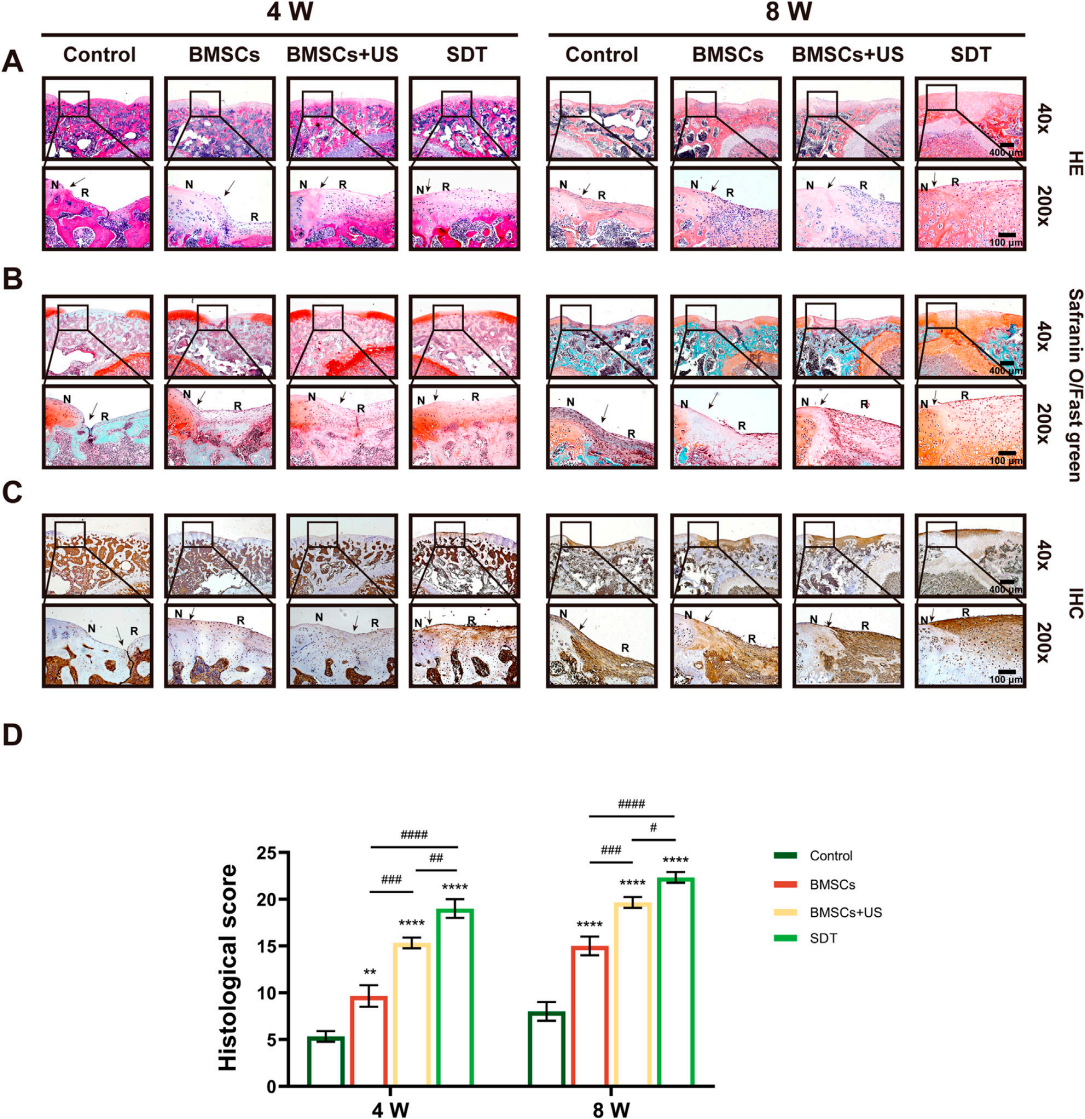
Histological observation of therapeutic effects of cartilage defect *in vivo*. **(A)** HE staining, **(B)** Safranin O/fast green staining, and **(C)** Immunohistochemical staining of tissue sections of knee joints with different magnifications. The black arrows show the edges of the repaired cartilage. N: normal cartilage; R: repaired cartilage. **(D)** The histological score of repaired cartilage. Mean ± SD, n = 3, * symbol compared with control group, ^**^ *p* < 0.01, ^***^ *p* < 0.001, and ^****^ *p* < 0.0001, and # symbol compared between groups, ^#^ *p* < 0.05, ^##^ *p* < 0.01, ^###^ *p* < 0.001, and ^####^ *p* < 0.0001.

In addition, the diseased sections underwent analysis by various staining techniques, including HE staining, safranin O/fast green staining, and immunohistochemistry staining, in order to assess the efficacy of cartilage repair. It found that the control group formed fibrous tissue in the defect area, creating a large residual gap with the original cartilage. A small number of round or oval depressed cells with the characteristics of chondrocytes and predominantly fibrous tissue were visible on the irregular surface of specimen sections from the BMSCs group. However, a large amount of newly formed cartilage tissue could be observed in specimen sections from the SDT group, whereas neo-cartilage tissue and a small amount of fibrous tissue could be observed in the BMSCs + US group (Figure 6A). In a similar vein, the SDT group exhibited the presence of tissue resembling hyaline cartilage, displaying favorable integration with the surrounding tissues as seen by safranin O/fast green staining in comparison to the other groups (Figure 6B). The technique of immunohistochemical staining was employed to ascertain the presence of *Col2a1* expression within the neo-cartilage tissue. As shown in Figure 6C, the repair area of the control group exhibited negative staining, whereas the group treated with BMSCs displayed a small amount of positive staining. However, the BMSCs + US group and SDT group stained strongly positive, indicating the formation of hyaline cartilage-like tissue. The histological grade was used to assess cartilage healing using histological investigation, hence corroborating the obtained findings (Figure 6D). The histology score in the SDT group at 8 weeks after surgery was 22.33 ± 0.82 scores, which exhibited a statistically significant increase compared to the other three groups.

## 4 Discussion

It has been reported that moderate ROS are important for cell growth and differentiation (Liu et al., 2023). During chondrogenesis, ROS are required for cell growth and differentiation of BMSCs (Lu et al., 2019). SDT has emerged as a potentially effective therapeutic modality that has garnered significant interest in recent years. It involves the use of ultrasound to induce sonoluminescence and activate sonosensitizers. The process of activation results in the generation of highly detrimental ROS, such as singlet oxygen and hydroxyl radicals. SDT has developed as a potential US-based treatment option for cartilage-related conditions, offering a new paradigm in cartilage therapy (Vahedi et al., 2021). In our study, we synthesized a good biodegradable, biocompatible sonosensitizer MOF-HMME-RGD to regulate chondrogenic differentiation of BMSCs for cartilage regeneration via the modulation of ROS.

The use of sonosensitizers in the context of SDT has been shown to effectively enhance the treatment of articular cartilage abnormalities (Cook et al., 2001; Xia et al., 2017; He et al., 2024). This approach has many advantages, including non-invasiveness, the ability to penetrate deep into the tissue, high therapeutic efficacy, and little occurrence of adverse effects. However, many sonosensitizers in clinical applications are still limited by the difficulties of non-biodegradation, poor water solubility, low efficient delivery, and potential biosafety issues (Nguyen Cao et al., 2023). We synthesized a good biodegradable, biocompatible sonosensitizer MOF-HMME-RGD, which significantly promoted the cell viability of BMSCs under ultrasound stimulation (Figure 3F). This is mainly due to the composition of MOF-HMME-RGD, including MOFs, hydrolysis products of endogenous hemoglobin HMME, and RGD peptide, which had good biocompatibility and biodegradability. On day 14, the expression levels of chondrogenic genes (*ACAN*, *SOX9*, and *Col2a1*) were found to be 1.21, 1.99, and 6.46-fold higher, respectively, in the MOF-HMME-RGD group (Figures 4C–E). The histological observations provided further confirmation of the regeneration of tissue resembling hyaline cartilage inside the intra-articular setting (Figures 6A–C), as well as the enhanced repair of cartilage defects after damage. The findings suggested that MOF-HMME-RGD exhibited good biocompatibility and low cytotoxicity, hence promoting cellular proliferation and differentiation towards the hyaline cartilage phenotype, which is crucial for chondrogenesis.

The regulation of ROS is an additional significant element that influences the process of chondrogenic differentiation (Bai et al., 2019). In recent years, accumulating studies indicated that ROS were signaling molecules, and a regulated basal level of ROS was necessary and had the advantage of cell functions, such as cell survival, proliferation, and differentiation (Li et al., 2017). ROS are essential for chondrogenesis, and ROS generation was increased in the early stage of chondrogenesis (Heywood and Lee, 2017). The MOF-HMME-RGD exhibited dual functionality as both sonosensitizers and ROS producers when subjected to ultrasonic stimulation. The percentage of cell apoptosis in MOF-HMME-RGD + US irradiation (5.54%) was low, indicating ROS generation by MOF-HMME-RGD nanoparticles was not cause more apoptosis. The T group, which did not have ultrasonic irradiation and thus lacked the ability to produce ROS, had little impact on the process of chondrogenic differentiation and cartilage regeneration. This conclusion is supported by the results obtained from GAG analysis (Figures 4A, B), qRT-PCR measurements (Figures 5C–E), and histological assessments (Figures 6A–C). For the TMU group exposed to ultrasound stimulation, it was observed that there was an increase in intracellular ROS in TMU (Figure 3C), which was associated with the capability to generate singlet oxygen generation detected by the chemical probe DPBF (Figure 3A). During the process of MSCs chondrogenic differentiation, the generated ROS as second messengers mediate the cellular alterations (glycolysis, oxidative metabolism), including alterations in molecules (proteins, nucleic acids, sugars, and lipids) that were essential for promoting differentiation (Li et al., 2017). As a result, MOF-HMME-RGD + SDT resulted in an upregulation of chondrogenic genes (*ACAN*, *SOX9* and *Col2a1*) (Figures 4C–E) and evidently promoted the cartilage regeneration (Figure 5B, Figures 6A–C). After treatment of NAC, which was a direct scavenger for ROS, a notable decrease in the accumulation of glycosaminoglycan, a cartilage-specific ECM component, was observed. Additionally, the expression of cartilage-specific genes was successfully inhibited, hence reducing the impact on chondrogenic differentiation. Our study suggested that ROS generation of MOF-HMME-RGD plays a critical role in BMSCs chondrogenesis and cartilage regeneration.

## 5 Conclusion

In summary, we synthesized a good biodegradable, biocompatible sonosensitizer MOF-HMME-RGD to regulate the chondrogenic differentiation of BMSCs for cartilage regeneration. The MOF-HMME-RGD NPs have the ability to generate a moderate level of ROS via the process of sonodynamic treatment. This leads to enhanced chondrogenic differentiation of BMSCs by promoting the accumulation of glycosaminoglycan, a particular ECM component found in cartilage. Furthermore, the upregulation of crucial chondrogenic genes, such as *ACAN*, *SOX9*, and *Col2a1*, occurs as a result. Moreover, the combination of transplanted BMSCs and SDT shows an augmented capacity for promoting cartilage regeneration in the context of repairing cartilage defects. This beneficial effect was seen after a duration of 8 weeks of therapeutic intervention. This study presents a synergistic technique that utilizes MOF nanoparticles as a basis for generating alternative sonosensitizers for the purpose of cartilage regeneration in conjunction with SDT.

## Data Availability

The original contributions presented in the study are included in the article/Supplementary material, further inquiries can be directed to the corresponding authors.
